# Synthesis and investigation of antiproliferative activity of Ru-NHC complexes against C6 and HeLa cancer cells

**DOI:** 10.55730/1300-0527.3418

**Published:** 2022-03-15

**Authors:** Ramazan PAŞAHAN, Mitat AKKOÇ, Şeyma YAŞAR, Tuğba KUL KÖPRÜLÜ, Şaban TEKİN, Sedat YAŞAR, İsmail ÖZDEMIR

**Affiliations:** 1 Department of Neurosurgery,, Faculty of Medicine, İnönü University, Malatya, Turkey; 2 Department of Property Protection and Security, Hekimhan Vocational College, Malatya Turgut Özal University, Malatya, Turkey; 3 Department of Biostatistics and Medical Informatics, Faculty of Medicine, İnönü University, Malatya, Turkey; 4 Experimental Medicine Application and Research Center, University of Health Sciences, İstanbul, Turkey; 5 Department of Chemistry, Faculty of Science and Arts, İnönü University, Malatya, Turkey

**Keywords:** C6, HeLa, antiproliferative activity, *N*-heterocyclic carbene, ruthenium, silver

## Abstract

The 2-methylpyridine, 2-diethylaminoethyl, and isopentyl linked a series of symmetric and unsymmetric benzimidazolium salts 2a–e were prepared and used in the synthesis of silver-N-heterocyclic carbene (NHC) complexes (3a–e). The Ru(II)-NHC complexes (4a–h) were synthesized via transmetalation reaction from 3a–e. 4a–h complexes were converted to Ru(II)-NHC.HCl complexes (5ah) by HCl solution of diethyl ether and characterized by different spectroscopic techniques such as ^1^H and ^13^C NMR, LC/MS-Q-TOF, FT-IR, elemental analysis, and melting point detection. We examined the effect of the structural difference of complexes on anticancer activity via different arenes and metal centers. Antiproliferative activity of 5a–h and 3a was tested against human cervix adenocarcinoma (HeLa) and rat glioblastoma (C6) cell lines by ELISA assay. The IC_50_ value of 5b, 5c and 5e complexes exhibited good cytotoxic activity than cisplatin on C6 (14.2 ± 0.5 mM; 16.2 ± 0.4 mM; 24.2 ± 0.7 mM, respectively) and HeLa (11.1 ± 0.5 mM; 13.7 ± 0.3 mM; 22.8 ± 0.8 mM, respectively) cell lines.

## 1. Introduction

Glioblastoma continues to be a lethal type of cancer with a low five-year survival rate despite total excision, radiotherapy, and chemotherapy [1]. Although researchers conducted various molecular and therapeutic studies, no significant progress was achieved in clinical practice. Therefore, animal glioma cell models are essential [2]. Cisplatin is one of the leading chemotherapy drugs used to treat several cancers. Although cisplatin is clinically effective in treating different types of cancer, its toxicity and the drug resistance of cells limit its use [3]. The discovery of Cisplatin has led to the idea that metal complexes may play an important role in chemotherapy. Exploring new types of drugs on medicinal applications remains a challenge to minimize toxic side effects, drug resistance, and inadequate solubility limitations of platinum-based drugs [4].

**Scheme 1:**
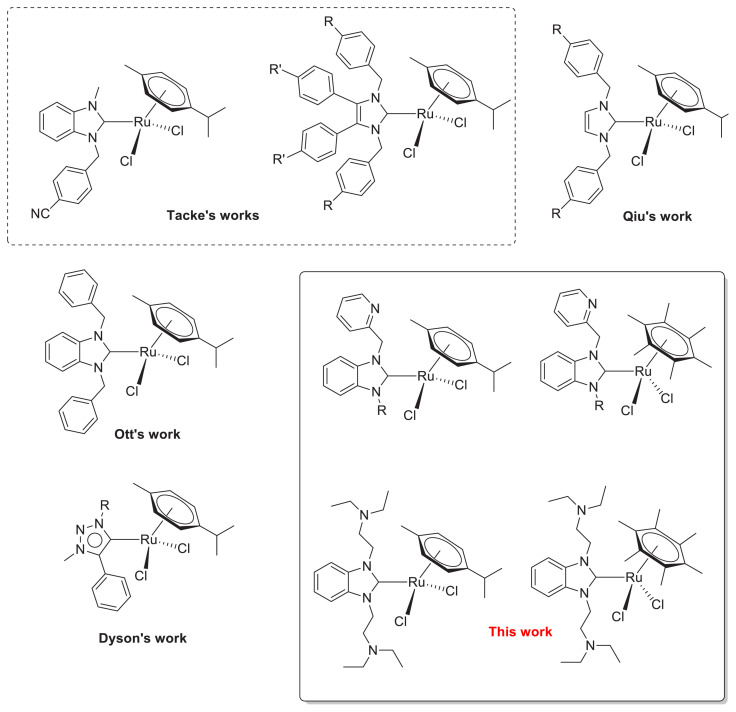
Structures of Ru-NHC complexes used against different cancer cell lines.

*N*-heterocyclic carbenes (NHCs) are easily synthesized, chemically modified, and exhibit superior properties ligands. The lipophilic end is essential in drug molecules, and to serve this lipophilic end on NHC, it needs to modify chemically. Thus, easy chemical modification of NHCs to serve lipophilic end in NHC-based drug molecules is significant. The NHCs can form a strong bond with the metal centers that lead to a more stable complex under moisture, heat, and air conditions. Due to these superior features, NHCs play an essential role in catalysis, biomedical applications, and functional material applications [5–14]. Studies have been focused on the biological application of Ag(I), Au(I), Ru(II), Rh(II), Pt(II), Pd(II), and Cu(I)-NHC complexes as antibacterial and anticancer agents [15–52]. Among synthesized NHC complexes, significant progress has been made with Ag-NHC and Au-NHC complexes on antibacterial and anticancer applications. Ag-NHC complexes remain therapeutically active longer than AgNO_3_, due to a slow speed deliver of Ag^+^ ions from high stable Ag-NHC complex [53]. Ruthenium-based complexes were used in medical applications due to less toxicity and are more capable of overcoming cancer cells’ resistance than Pt-based drugs [54–58]. Benefits of Ru complexes in biological applications were reported by different groups [59–64]. The most prominent feature of ruthenium in these studies is that it imitates iron element in binding to biological molecules such as albumin and transferrin [57,64–66]. These ruthenium complexes have been designed for DNA-targeting, but ruthenium complexes weakly interact with DNA compared to analogous Pt complexes [57,67]. However, DNA targeting is unnecessary for bioactivity because NAMI-A shows an extracellular mechanism to inhibit cancer cell motility [68]. KP1019 shows mild in vitro cytotoxicity without targeting the DNA of cancer cells [69]. Different research teams have made intensive investigations on anticancer applications of ruthenium complexes [70–73]. Burgos et al. investigated antioxidant/prooxidant activity and toxicity of some of the ruthenium-arene complexes. They concluded that Ru(II) arene complexes behave as oxidants at low concentrations and as prooxidants at high concentrations. However, they reported that the ruthenium complexes could not negatively affect the Zebrafish embryos. Therefore, Ru(II)-arene complexes can be considered nontoxic [74–75]. Due to the low toxicity of NAMI-A, AziRu, and KP1019 ruthenium complexes and their ability to overcome the resistance of cancer cells to drugs, their phase II clinical trials have been started [76–81]. The antiproliferative effects of six Ru-NHC complexes against MCF-7 and Caki-1 cancer cell lines were investigated by Tacke et al. (Scheme 1). They found that these complexes showed lower and better activity than cisplatin on Caki-1 and MCF-7 cells. They stated that the reason behind these results was influenced substituents in the imidazole group. Ott et al. sythesized a series of benzimidazole-based Ru(II)-NHC-(-cymene) Cl_2_ complexes and investigated their behavior on MCF-7 and HT-29 (Scheme 1) [71]. The ruthenium complex, which bears benzyl group as *N*-substituent on the NHC, showed pronounced activities on MCF-7 and HT-29 in low micromolar concentrations (Scheme 1) [71].

Considering cisplatins limitation due to the solubility problem, there is intense interest in synthesizing different water-soluble metal complexes. A fine-tune hydrophilic moiety can provide the water solubility of complexes on the ligands [82–86]. Recently, our group reported cytotoxic properties of the Ag-NHC complexes on HeLa, HT-29, and L929 cell lines [87]. Among the NHCs, benzimidazole-based silver, gold and ruthenium-NHC complexes have been studied intensely due to the benzimidazole structure being a component of many biological structures [88–94]. In our previous study, Ru-NHC complexes showed good antiproliferative activity on Caco-1 and MCF-7 cell lines [16]. Encouraged by these results, we thought it would be helpful to examine the anticancer activities of the similar ruthenium complexes against different types of cancer cell lines to determine the affinity between them.

**Scheme 2:**
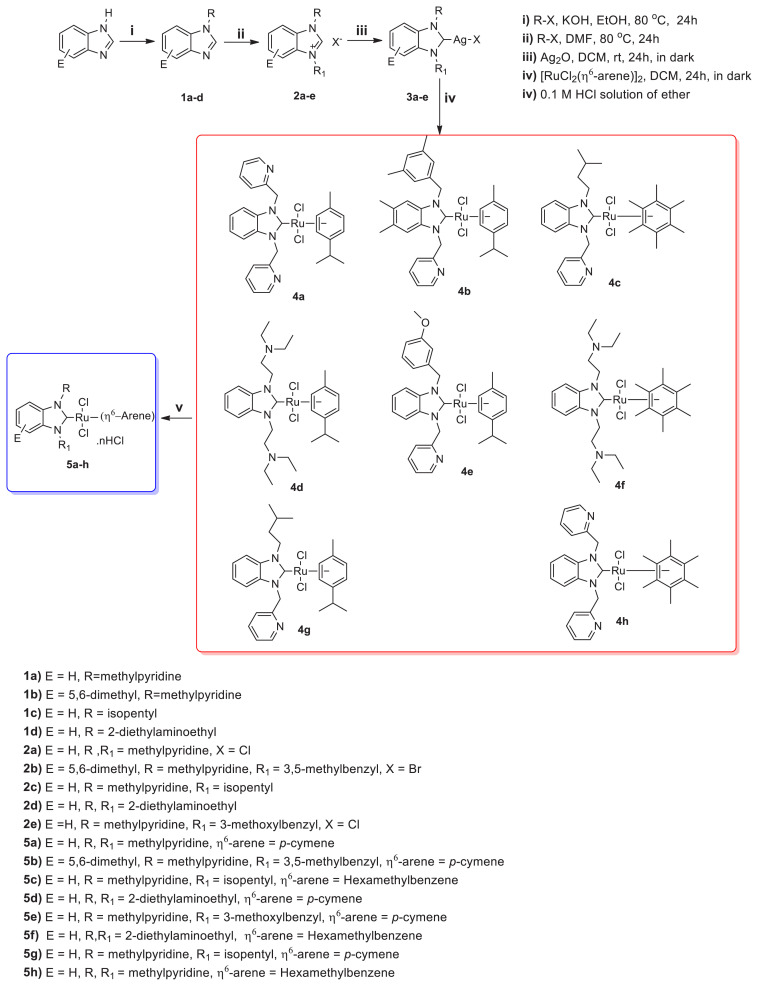
Synthesis pathway of **1a–d**, **2a–e**, **3a–e**, **4a–h**, and **5a–h**.

Herein, we synthesized and investigated the anticancer activity of eight Ru-NHC complexes and one of the Ag-NHC complexes with good lipophilic and hydrophilic properties on C6 and HeLa cell lines by a proliferation BrdU enzyme-linked immunosorbent assay (ELISA) (Scheme 2). These water-soluble Ru-NHC complexes displayed pronounced anticancer activity on C6 and HeLa cancer cells.

## 2. Experimental

### 2.1. General considerations

All reactions were performed under Ar (argon) gas. Ag-NHC and Ru-NHC complexes were synthesized under the exclusion of light. ^1^H and ^13^C Nuclear Magnetic Resonance (NMR) analysis were performed by a Bruker Avance III HD 300 and 400 MHz NMR spectrometer. The FT-IR analyses were performed with a PerkinElmer Spectrum 100 GladiATR FT/IR spectrometer. Elemental analyses were recorded by a LECO, CHNS-932 elemental analyzer. The LC/MS-IT-TOFF (ESI) electrospray ionization CH_3_CN/CHCl_3_. Absorbances were measured by a BioTek-Epoch microplate reader.

### 2.2. Cell culture

The human cervix adenocarcinoma (HeLa) and rat glioblastoma (C6) cell lines were grown as in the relevant literature [87]. All assays were performed in triplicate.

### 2.3. BrdU cell proliferation ELISA (BCPE)

BrdU cell proliferation ELISA (Roche, USA) kit based on the detection of BrdU incorporation during DNA synthesis was used to measure the compounds’ antiproliferative activity. Cell suspensions containing 3 × 10^3^ cells in 100 mL were pipetted into the wells of 96-well cell culture plates (COSTAR, Corning, USA). The test compounds and positive control (Cisplatin, Sigma, Germany) were prepared as in the relevant literature [87]. Eight different concentrations of the complexes were used. The concentration of complexes and cisplatin was serially increased and their effect on growth inhibition of cancer cells was observed.

### 2.4. Synthesis

#### 2.4.1. Synthesis of N-alkylbenzimidazole (1a–d) and NHCs (2a–e)

*N*-alkylbenzimidazoles and NHC precursors were synthesized according to the related literature (Scheme 2) [15, 16, 95–97]. **2a**, **2c**, and **2e** were synthesized according to the literature [16].

#### 2.4.2. 1-(methylpyridine)-3-(3,5-dimethylbenzyl)-5,6-dimethylbenzimidazolium chloride, 2b

Compound **2b** was synthesized by the reaction of **1b** (1 mmol) and 3,5-dimethylbenzyl bromide (1.1 mmol) [16]. The solid was washed with hexane and dried (0.3 g, 85%). M.p: 254 °C. ^1^H NMR (300 MHz, CDCl_3_) δ = 11.64 (s, 1H, NC**H**N), 8.43 (d, *J* = 4.8 Hz, 1H, CH_2_C_6_**H_4_**N), 7.84–6.88 (m, 8H, C_6_**H_2_**(CH_3_)_2_-5,6, CH_2_C_6_**H_4_**N and C_6_**H_3_**(CH_3_)_2_-3,5), 5.96 (s, 2H, C**H_2_**C_6_H_3_(CH_3_)_2-_3,5), 5.58 (s, 2H, C**H_2_**C_6_H_4_N), 2.29 (s, 3H, C_6_H_2_(C**H_3_**)_2_-5,6), 2.26 (s, 3H, C_6_H_2_(C**H_3_**)_2_-5,6), 2.20 (s, 6H, C_6_H_3_(C**H_3_**)_2_-3,5).^13^C NMR (75 MHz, CDCl_3_) δ = 152.7, 149.4, 142.7, 139.0, 137.7, 137.2, 137.1, 132.6, 130.7, 130.4, 129.7, 125.5, 123.8, 114.0, 113.0, 52.2, 51.2, 21.2, 20.7, 20.6.

##### 2.4.2.1. 1,3-bis-(2-diethylamino)ethyl)benzimidazolium chloride, 2d

**2d** was synthesized as brown crystals (0.31 g, 88%) by the reaction of **1d** (1 mmol) and 2-(diethylamino)ethyl chloride (1.1 mmol). M.p: 151 °C. ^1^H NMR (300 MHz, CDCl_3_) δ = 11.08 (s, 1H, NC**H**N), 8.88 and 7.63 (dd, *J* = 3.0 Hz, 4H, C_6_**H_4_**), 4.78 (t, *J* = 6.3 Hz, 4H, C**H_2_**CH_2_N(C_2_H_5_)_2_), 3.08 (t, *J* = 6.3 Hz, 4H, CH_2_C**H_2_**N(C_2_H_5_)_2_), 2.67 (q, *J* = 7.2 Hz, 8H, CH_2_CH_2_N(C_2_**H_5_**)_2_), 0.94 (t, *J* = 7.2 Hz, CH_2_CH_2_N(C_2_**H_5_**)_2_). ^13^C NMR (75 MHz, CDCl_3_) δ = 144.1, 131.2, 126.9, 113.4, 51.5, 46.9, 45.6, 11.2.

#### 2.4.3. Synthesis of Ag-NHC complexes (2a–e)

Complexes **2a–e** were prepared as in the relevant literature [16]. Detail of the complexes **3a**, **3c**, and **3e** can be found in the related literature [16].

##### 2.4.3.1. Chloro[1-(methylpyridine)-3-(3,5-dimethylbenzyl)-5,6-dimethylbenzimidazol-2-yliden] silver(I), 3b

Complex **3b** was synthesized as brown powder solid (0.37 g, 75%): M.p: 200 °C. ^1^H NMR (400 MHz, CDCl_3_) δ = 8.62 (d, *J* = 2 Hz, 1H, C_5_**H_4_**N), 7.69–6.86 (m, 8H, C_6_**H_2_**-(CH_3_)_2_-5,6 and C_5_**H_4_**N), 5.71 (s, 2H, C**H_2_**C_6_H_3_-(CH_3_)_2_-3,5), 5.51 (s, 2H, C**H_2_**C_5_H_4_N), 2.32–2.29 (s, 12 H, CH_2_C_6_H_3_**-(CH_3_)_2_**-3,5, C_6_H_2_**-(CH_3_)_2-_**5,6). ^13^C NMR (100 MHz, CDCl_3_) δ = 155.0, 149.8, 138.8, 137.3, 134.9, 134.0, 132.5, 130.2, 124.8, 123.3, 121.7, 112.4, 112.2, 55.0, 53.2, 21.3, 20.4 ppm. HRMS (ESI) m/z [M + H]^+^ was calculated for C_24_H_26_N_3_Ag: 437.0657 and found 437.0671.

##### 2.4.3.2. Chloro[1,3-Bis-(2-(diethylamino)ethyl)benzimidazol-2-yliden] silver (I) dihidrochloro, 3d

Complex **3d** was synthesized as brown solid (0.40 g, 88%). M.p: 205 °C. ^1^H NMR (400 MHz, DMSO-d_6_) δ = 7.80–7.41 (m, 4H, C_6_H_4_), 4.46 (t, *J* = 6 Hz, 4H, C**H_2_**CH_2_NCH_2_CH_3_), 2.80 (t, *J* = 6 Hz, 4H, CH_2_C**H_2_**NCH_2_CH_3_), 2.47 (t, *J* = 8 Hz, 8H, CH_2_CH_2_NC**H_2_**CH_3_), 0.83 (t, *J* = 6 Hz, 12H, CH_2_CH_2_NCH_2_C**H_3_**). ^13^C NMR (100 MHz, DMSO-d_6_) δ = 133.8, 124.1, 112.5, 52.9, 47.9, 47.4, 12.4.

#### 2.4.4. Synthesis of Ru-NHC complexes

The Ru-NHC complexes were synthesized as in the relevant literature [16, 98]. Details of the complexes **5a**, **5c**, and **5e** can be found in related literature [16].

##### 2.4.4.1. [1-(methylpyridine)-3-(3,5-dimethylbenzyl)-5,6-dimethylbenzimidazole-2-yliden](h_6_-****-cymene)ruthenium (II) dichloride.HCl, 5b (C_34_H_39_N_3_Cl_2_Ru. HCl)

Complex **5b** was synthesized in an analogous manner to complex **5a** with use of **3b** (1 mmol), which gave complex **5b** as dark orange solid (1.17 g, 84%). M.p.: 229 °C. u_C-N_ = 1413.50 cm^–1^. ^1^H NMR (300 MHz, D_2_O) δ = 8.98 (m, 1H, CH_2_C_5_**H_4_**N), 7.60–4.95 (m, 16H, CH_2_C_5_**H_4_**N, C_6_**H_2_**(CH_3_)_2_-5,6, CH_2_C_6_**H_4_**(CH_3_)_2_-3,5, C**H_2_**C_5_H_4_N, CH_2_C_6_**H_4_**(CH_3_)_2_-3,5, C**H**(-cymene)), 2.36 (m, 1H, C**H**(i-pr)(-cymene)), 1.91–1.46 (m, 15H, C_6_H_2_(C**H_3_**)_2_-5,6, CH_2_C_6_H_4_(C**H_3_**)_2_-3,5, C**H_3_**(-cymene)), 0.82 (m, 6H, C**H_3_**(i-Pr)). ^13^C NMR (75 MHz, D_2_O) δ = 187.7, 155.4, 137.9, 136.3, 133.4, 132.7, 132.3, 124.2, 110.9, 102.7, 87.0, 85.5, 65.9, 34.4, 31.1, 22.7, 20.5, 19.3, 17.8, 14.0. HRMS (*m/z*, LCMS-QTOF (ESI)): 599.1498 [M^+^ - Cl], calcd. for C_32_H_35_ClN_3_Ru 599.1641. Anal. calcd. for C_34_H_40_N_3_Cl_3_Ru: C, 58.49; H, 5.78; N, 6.02. Found: C, 58.66; H, 5.84; N, 6.12

##### 2.4.4.2. Dichloro[1,3-bis(2-diethylamino)ethyl)benzimidazol-2-yliden](p-cymene)ruthenium(II).2HCl, 5d (C_29_H_46_N_4_ Cl_2_Ru. 2HCl)

Complex **5d** was synthesized in an analogous manner to complex **5a** with use of **3d** (1 mmol), which gave complex **5d** as a dark red powder (1.04 g, 75%). M.p.: 238 °C. u_C-N_ = 1470.4 cm^-1^. ^1^H NMR (300 MHz, D_2_O) δ = 7.64–7.42 (m, 4H, C_6_H_4_), 5.89–5.34 (m, 4H, CH(p-cymene)), 4.99–2.85 (m, 16H, CH_2_CH_2_NCH_2_CH_3_, CH_2_CH_2_NCH_2_CH_3_, CH_2_CH_2_NCH_2_CH_3_), 2.42 (p, 1H, CH(i-pr)(p-cymene)), 2.03 (s, 3H, CH_3_(p-cymene)), 1.44–0.68 (m, 16H, CH_3_(i-Pr), CH_2_CH_2_NCH_2_CH_3_). ^13^C NMR (75 MHz, D_2_O) δ = 183.5, 135.3, 134.5, 134.0, 128.1, 124.6, 124.4, 113.1, 110.9, 110.3, 94.9, 87.5, 86.3, 85.5, 84.5, 81.1, 58.3, 57.3, 50.4, 48.6, 47.9, 46.2, 43.9, 42.2, 30.2, 23.2, 21.3, 18.9, 17.1, 11.1. HRMS (*m/z*, LCMS-QTOF (ESI)): 587.2454 [M^+^ - Cl], calcd. for C_29_H_46_ClN_4_Ru 587.2454. Anal. calcd. for C_29_H_48_N_4_Cl_4_Ru: C, 50.07; H, 6.96; N, 8.05. Found: C, 50.19; H, 7.10; N, 8.19

##### 2.4.4.3. Dichloro[1,3-bis(2-diethylamino)ethyl)benzimidazol-2-yliden](hexamethylbenzene) ruthenium(II).2HCl, 5f (C_31_H_50_N_4_Cl_2_Ru. 2HCl)

Complex **5f** was synthesized an in an analogous manner to complex **5a** with use **3d** (1 mmol), which gave complex **5f** as a dark red powder (1.27 g, 88%). M.p.: 205 °C. u_C-N_ = 1457.08 cm^-1^. ^1^H NMR (300 MHz, D_2_O) δ = 7.93–7.42 (m, 4H, C_6_H_4_), 4.84 (t, *J* = 7.2 Hz, 4H, CH_2_CH_2_NCH_2_CH_3_), 3.54 (m, 2H, CH_2_CH_2_NCH_2_CH_3_), 3.31 (m, 8H, CH_2_CH_2_NCH_2_CH_3_), 1.88 (s, 18H, C_6_(CH_3_)_6_), 1.25 (t, *J* = 7.5 Hz, 12H, CH_2_CH_2_NCH_2_CH_3_). ^13^C NMR (75 MHz, D_2_O) δ = 193.5, 134.6, 130.9, 128.1, 124.5, 113.1, 110.5, 95.7, 50.4, 49.1, 48.7, 47.9, 43.6, 41.6, 15.3, 15.2, 14.9, 8.3, 8.0. HRMS (*m/z*, LCMS-QTOF (ESI)): 615.2764 [M^+^ - Cl], calcd. for C_31_H_50_ClN_4_Ru 615.2767. Anal. calcd. for C_31_H_52_N_4_Cl_4_Ru: C, 51.45; H, 7.24; N, 7.74. Found: C, 51.56; H, 7.40; N, 7.88.

##### 2.4.4.4. Dichloro[1,3-bis(methylpyridine)benzimidazol-2-yliden]hexamethylbenzene ruthenium(II).2HCl, 5h (C_31_H_34_N_4_Cl_2_Ru. 2HCl)

Complex **5h** was synthesized in an analogous manner to complex **5a** with use **3a** (1 mmol) gave complex **5h** as a dark red powder (1.20 g, 85%). M.p: 197 °C. u_C-N_ = 1438.50 cm^−1^.^1^H NMR (300 MHz, D_2_O) δ = 8.70–6.93 (m, 12H, CH_2_C_6_**H_4_**N and C_6_**H_4_**), 6.76 (d, *J* = 3.9 Hz, 1H, CH_2_C_6_**H_4_**N _2_), 5.85 (m, 2H, C**H_2_**C_6_H_4_N), 1.87 (s, 18H, C_6_(C**H_3_**)_6_). ^13^C NMR (75 MHz, D_2_O) δ = 193.5, 156.9, 155.2, 153.6, 149.2, 146.4, 140.2, 139.9, 134.5, 134.2, 126.0, 125.7, 124.0, 123.6, 122.7, 113.5, 111.4, 110.4, 98.2, 51.6, 51.4, 50.3, 15.1. Anal. calcd. for C_31_H_36_N_4_Cl_4_Ru: C, 52.62; H, 5.13; N, 7.92. Found: C, 52.74; H, 5.31; N, 8.01.

## 3. Results and discussion

**Figure 1:**
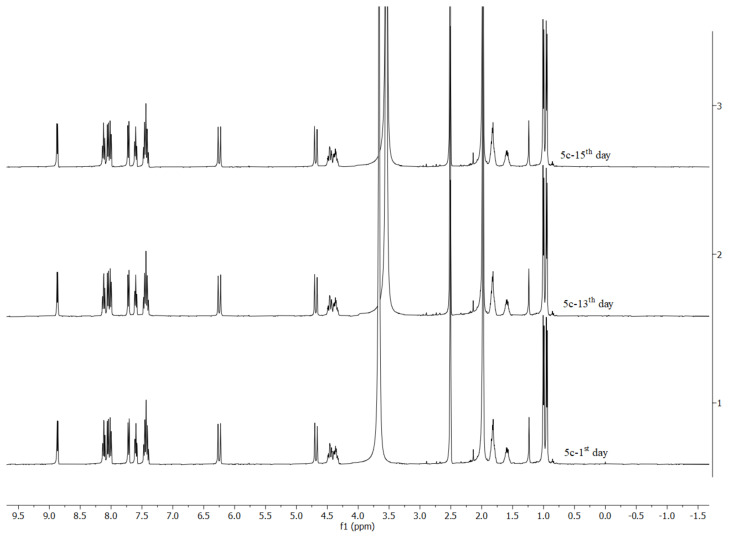
The stability test of complex **5c** in DMSO-d_6_ during 15 days by ^1^H NMR spectroscopy.

The synthesis pathway for the Ag-NHC and Ru-NHC complexes is presented in Scheme 2. The Ag-NHC complexes **3b** and **3d** were synthesized in good yields of 75% and 88%, respectively, by the reported procedure [95–97]. The Ru-NHC complexes were synthesized by transmetalation reaction in DCM from **3a–e** complexes, respectively. Transmetalation is one of the most general methods for preparing a wide range of transition metal complexes due to its mild reaction conditions and generating air-stable intermediates. The transmetalation reaction of Ag(I)-NHC with corresponding Ru(II)-arene dimer under the exclusion of light at room temperature led to corresponding Ru-NHC complexes. The **5b**, **5d**, **5f**, and **5h** (Ru-NHC.nHCl) complexes were synthesized in moderate to good yields of 84%, 75%, 88%, and 85%, respectively, by adding HCl-diethyl ether solution to the DCM solution of the **4b, 4d, 4f**, and **4h** complexes. Synthesized complexes are well soluble in polar solvents such as H_2_O, DCM, DMF, DMSO, CH_3_OH. The stability of **5c**, **5e**, and **5g** complexes was tested by ^1^H NMR spectroscopy and it was seen that Ru(II)-NHC complexes showed high stability without structural decomposition against oxygen and moisture during two weeks (Figures 1, S1, and S2). Structural descriptions of the complexes were performed by ^1^H NMR, ^13^C NMR, HRMS (Figure S3–S10), elemental analysis, and melting point determination.

The resonance of the C2 proton and C2 carbon of **2b** and **2d** in the ^1^H and ^13^C NMR were observed at 11.64, 11.08 152.7, and 144.1 ppm in CDCl_3_, respectively. The loss of the C2 proton in ^1^H NMR and downfield shift of the C2 carbon to a new area in ^13^C NMR spectra of Ag-NHC indicate the formation of Ag-NHC complexes. However, the C2 carbon of **3b** and **3d** was not observed in ^13^C NMR spectra. We think the fast interconversion in the NMR time scale between the mono-carbene and bis-carbene structures causes the C2 carbon to be invisible in ^13^C NMR spectra. According to Lin and coworkers [98], since the carbene-silver bond is labile in solution, the resonance of the carbene carbon, which is expected to be observed in the ^13^C NMR, may not be observed. In the ^13^C NMR spectrum of **5d**, **5d**, **5f**, and **5h** complexes, the carbene carbons dramatically shift downfield to 187.7, 183.5, 193.5, and 193.5 ppm in the ^13^C NMR spectra indicating the formation of **5d**, **5d**, **5f**, and **5h** complexes, respectively. The LCMS-QTOF spectra were verified in the **5b, 5d**, and **5h** complexes. The calculated and experimental LCMS-QTOF values are compatible with each other and confirm the proposed complex structures. NMR spectra of newly synthesized compounds and HRMS spectra are given in the supporting information part.

**Figure 2:**
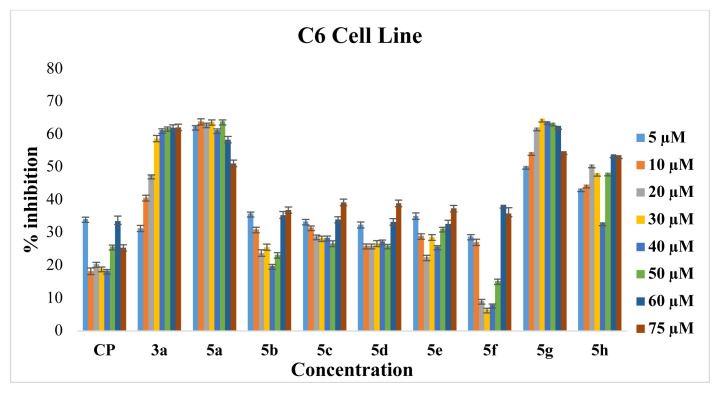
The antiproliferative effects of **3a** and **5a–h** complexes on C6 cells analyzed by BCPE.

**Figure 3:**
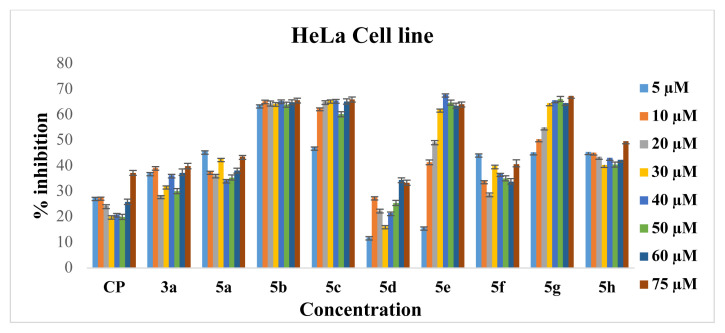
The antiproliferative effects of **3a** and **5a–h** complexes on HeLa cells analyzed by BCPE.

Cytotoxic activities of synthesized Ru-NHC complexes were investigated on C6 and HeLa cell lines. Figures 2 and 3 and Table present the inhibition and IC_50_ values of **3a** and **5a–h** on C6 and HeLa cell lines, respectively. The synthesized Ru-NHC complexes are both soluble in H_2_O and stable in the DMSO-d_6_ over the testing period. The Ru(II)-NHC and Ag(I)-NHC complexes except showed moderate (**5d**), good (**3a**, **5a**, **5f**, **5h**) and excellent (**5b**, **5c**, **5e**, **5g**) activity when compared to cisplatin, which exhibited an IC_50_ value of 136 ± 0.74 mM and 126 ± 0.57 mM against C6 and HeLa, respectively. However, when the structures of the complexes are examined, it is seen that structural differences cause antiproliferative activity differences in different cancer cell types. For example, *N*-substituents on the NHC and type of arene group led to a difference in ruthenium complexes antiproliferative activity against both cancer cell lines. The complexes, which are bearing asymmetric *N*-heterocyclic carbene ligand, showed excellent antiproliferative activity; methylpyridine and 2-diethylaminoethyl groups provide a moderate antiproliferative activity while 3-methoxybenzyl, 3,5-dimethylbenzyl, 2-aminoethyl and isopentyl groups led to high (IC_50_ for HeLa: **5b**, 11.1 ± 0.5; **5c**, 13.7 ± 0.3; **5e**, 22.8 ± 0.8; **5g**, 17.3 ± 0.8; **5h**, 46.8 ± 0.5; IC_50_ for C6: **5b**, 14.2 ± 0.5; **5c**, 16.2 ± 0.4; **5e**, 24.2 ± 0.7; **5g**, 37.3 ± 0.9; **5h**, 90.6 ± 0.7 **m**M) antiproliferative activity. However, the displacement of the *p-*cymene arene group by hexamethyl benzene increases the antiproliferative activity of **5f**. Complexes **5c** and **5g**, which are structurally identical except the arene group, showed a difference in the antiproliferative activity on C6 and HeLa cells. In both cell lines, the **5c** complex showed much better antiproliferative activity than the **5g** complex. The situation in the antiproliferative activities of the **5a** and **5h** complexes also changes in line with this trend, and complex **5h** showed slightly better activity than complex **5a**. The type of arene ligand also affected the antiproliferative activities of Ru(II)-NHC complexes because of the s-donor-p-acceptor ability of arene’s and NHC’s [60, 99]. This work gives us some useful info about the effect of the metal center’s genus on antiproliferative activity. Complex **3a** is an analog of complex **5a** except for the metal genus. When the antiproliferative activities of these two complexes are compared in the same cancer cells, it is seen that complex **5a** has shown better activity. This result may be an indicator of how important the metal genus is in anticancer activity.

The exact mode of action (MOA) of Ru-based complexes is unknown; as a result, a lot of Ru-containing drugs are still under development. Ru-complexes can imitate the iron-binding to serum transferrin which solubilizes and transports iron in the plasma thereby inhibiting their toxic delivery of iron. Additionally, numerous oxidation states, kinetics and different MOA provide many advantages over Pt-based complexes. For example, at physiological conditions, the Ru is known to be stable II, III, and IV oxidation states. The slow ligand exchange rates of the Ru-compounds make them suitable for biological applications. The good cytotoxicity of the Ru-complexes is due to their strong binding with DNA. Studies showed that some Ru-compounds could produce mutagenic effects, inhibit the replication of DNA, induce SOS repair, and decrease the synthesis of RNA thereby suggesting a DNA interaction [100]. In addition, according to our previous work [16], molecular docking calculations of similar Ru-NHC complexes showed anticancer activity by binding to DNA.

These observations point out that (a) the modification or fine-tune of the steric and electronic properties of NHCs through the *N*-substituents is crucial, (b) the arene type and metal center genus have a significant influence on the antiproliferative activity of complexes, and (c) complexes have properties that facilitate their cellular uptake into cells.

## 4. Conclusions

A series of Ru-NHC complexes have been prepared, spectroscopically characterized, and antiproliferative activity of complexes was examined on C6 and HeLa cells by a proliferation BrdU ELISA assay. The cytotoxic activities of complex **5b** and **5c** on C6 and HeLa cell lines are 7–9 times better than those of cisplatin and 2–10 times better than their analogous ruthenium complexes. Complexes **5b**, **5c**, and **5e** have shown excellent low micromolar activity against C6 and HeLa cell lines. Additionally, other ruthenium and silver complexes have shown better activity on every concentration than cisplatin except complex **5d**. The lower IC_50_ values of the Ru-NHC complexes **5b**, **5c**, **5e** are most likely to be attributed to the better solubility in H_2_O due to asymmetric NHCs. In addition, better solubility of complexes in H_2_O enhanced cellular uptake of complexes into the cell. This finding indicates that type of *N*-substituents on NHC and arene groups may improve the activity and selectivity. In this manner, the availability of effective drugs will lead to powerful medical treatment, and consequently, the number of surgical treatments will decrease, and life processes will increase.

## Supporting Information

Figure S1 The stability test of complex **5e** in DMSO-d_6_ during 14 days by ^1^H NMR spectroscopy.

Figure S2 The stability test of complex **5g** in DMSO-d_6_ during 14 days by ^1^H NMR spectroscopy.

Figure S3 The ^1^H NMR and ^13^ NMR spectra of **2b**.

Figure S4 The ^1^H NMR and ^13^ NMR spectra of **2d**.

Figure S5 The ^1^H NMR and ^13^ NMR spectra of **3b**.

Figure S6 The ^1^H NMR and ^13^ NMR spectra of **3d**.

Figure S7 The ^1^H NMR, ^13^ NMR and HRMS spectra of **5b**.

Figure S8 The ^1^H NMR, ^13^ NMR and HRMS spectra of **5d**.

Figure S9 The ^1^H NMR, ^13^ NMR and HRMS spectra of **5f**.

Figure S10 The ^1^H NMR and ^13^ NMR spectra of **5h**.

## Figures and Tables

**Table:** The IC_50_ values of **3a** and **5a–h** on C6 and HeLa cell lines.

IC_50_ (mM)	C6	HeLa
**3a**	106.1 ± 0.2	126.6 ± 0.6
**5a**	97 ± 0.9	90.6 ± 0.2
**5b**	14.2 ± 0.5	11.1 ± 0.5
**5c**	16.2 ± 0.4	13.7 ± 0.3
**5d**	159.1 ± 0.4	122 ± 0.4
**5e**	24.2 ± 0.7	22.8 ± 0.8
**5f**	95.1 ± 0.4	89.7 ± 1.0
**5g**	37.3 ± 0.9	17.3 ± 0.8
**5h**	90.6 ± 0.7	46.8 ± 0.5
**Cisplatin**	136 ± 0.7	126 ± 0.6

The IC_50_ (**m**M) ± S.E. ^[a]^S. E. = Standard error

## Data Availability

The data that support the findings of this study are available from the corresponding author upon reasonable request.
