# Supplementary figures and images for: Synthesis and investigation of antiproliferative activity of Ru-NHC complexes against C6 and HeLa cancer cells
Source: Turk J Chem. 2022 Mar 15;46(4):1097–109. doi: 10.55730/1300-0527.3418 (PMC10395682; doi:10.55730/1300-0527.3418)

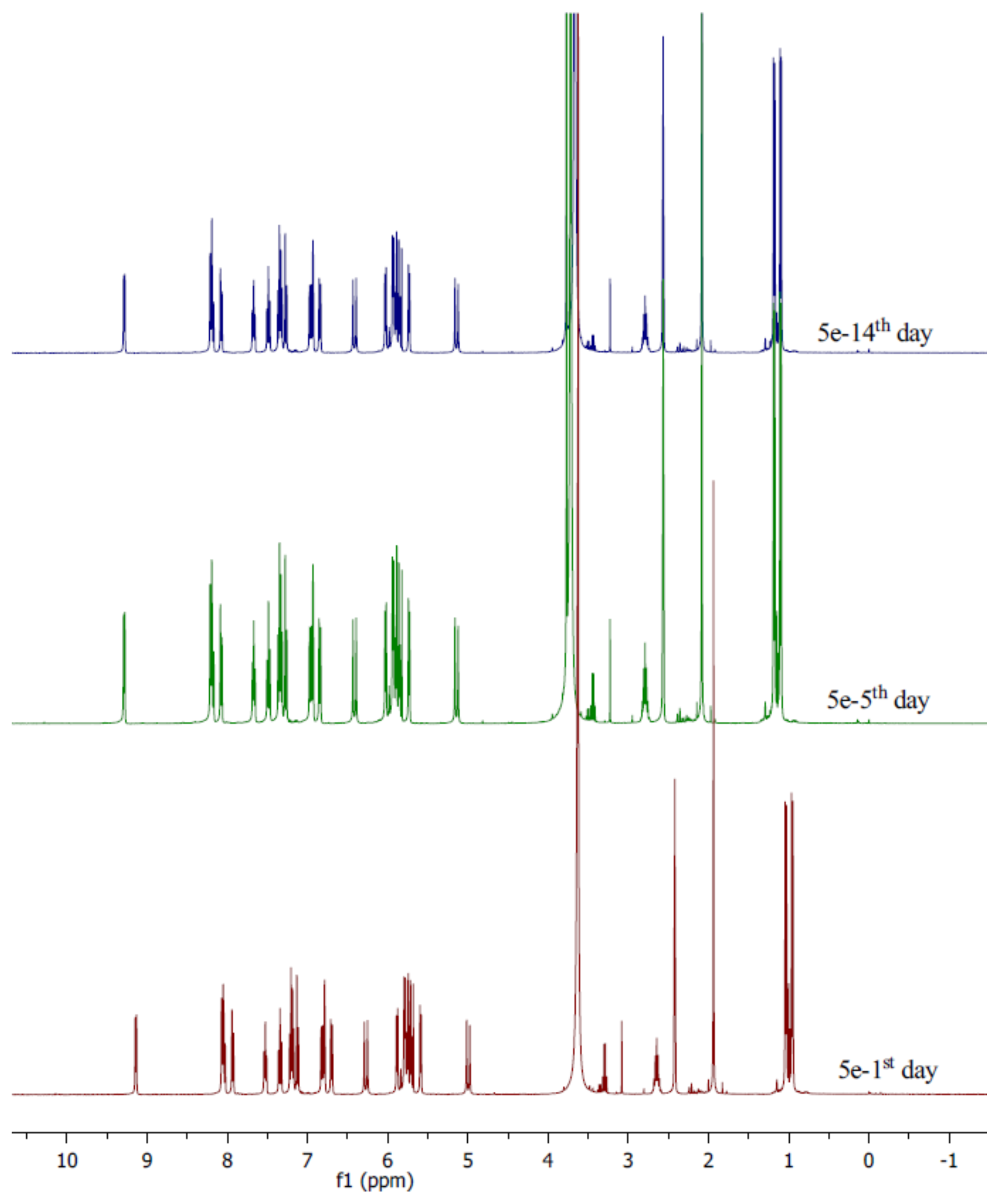

Supplement: Figure S1 — The stability test of complex 5e in DMSO-d6 during 14 days by 1H NMR spectroscopy. [file turkjchem-46-4-1097s1.tif]

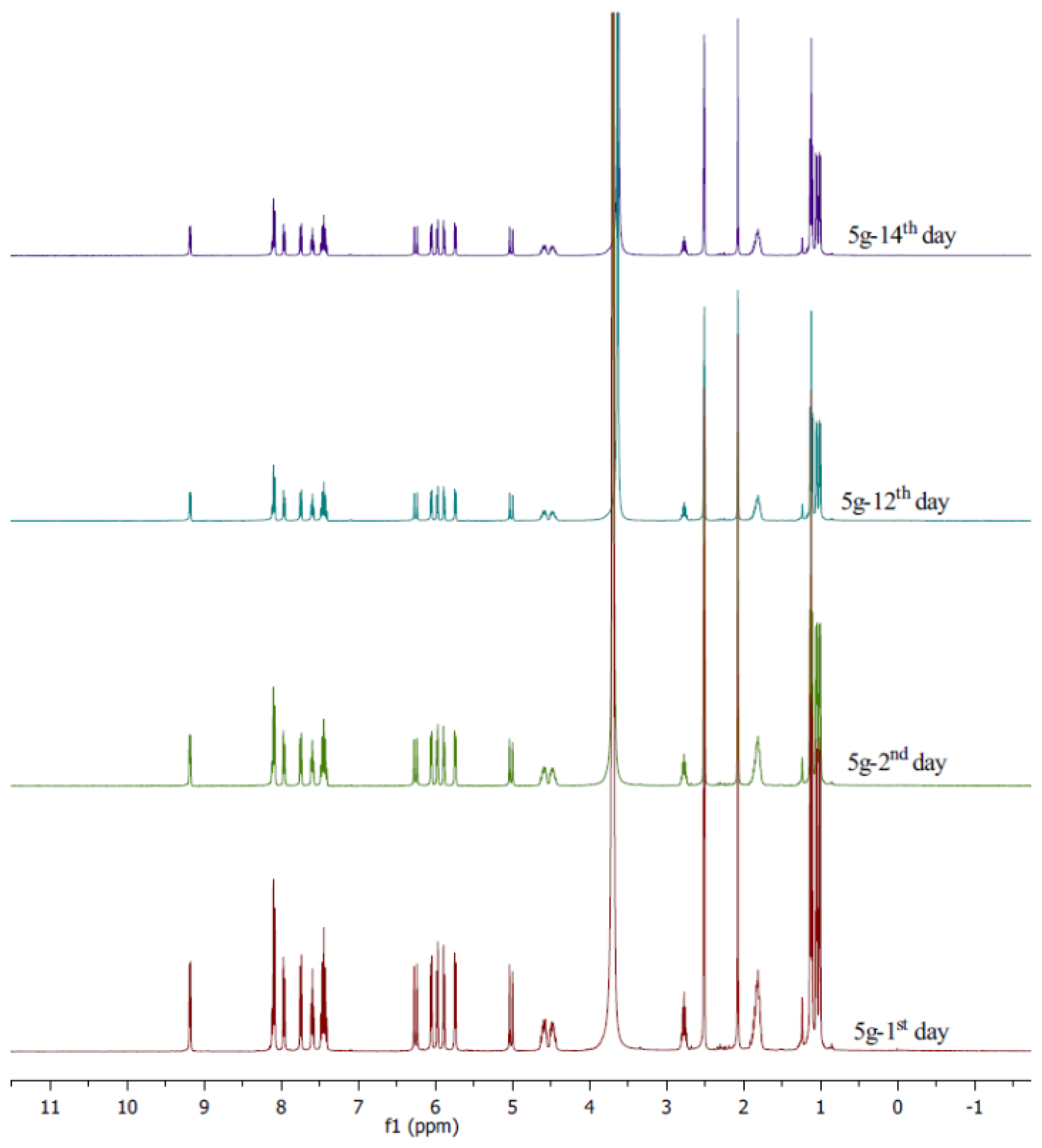

Supplement: Figure S2 — The stability test of complex 5g in DMSO-d6 during 14 days by 1H NMR spectroscopy. [file turkjchem-46-4-1097s2.tif]

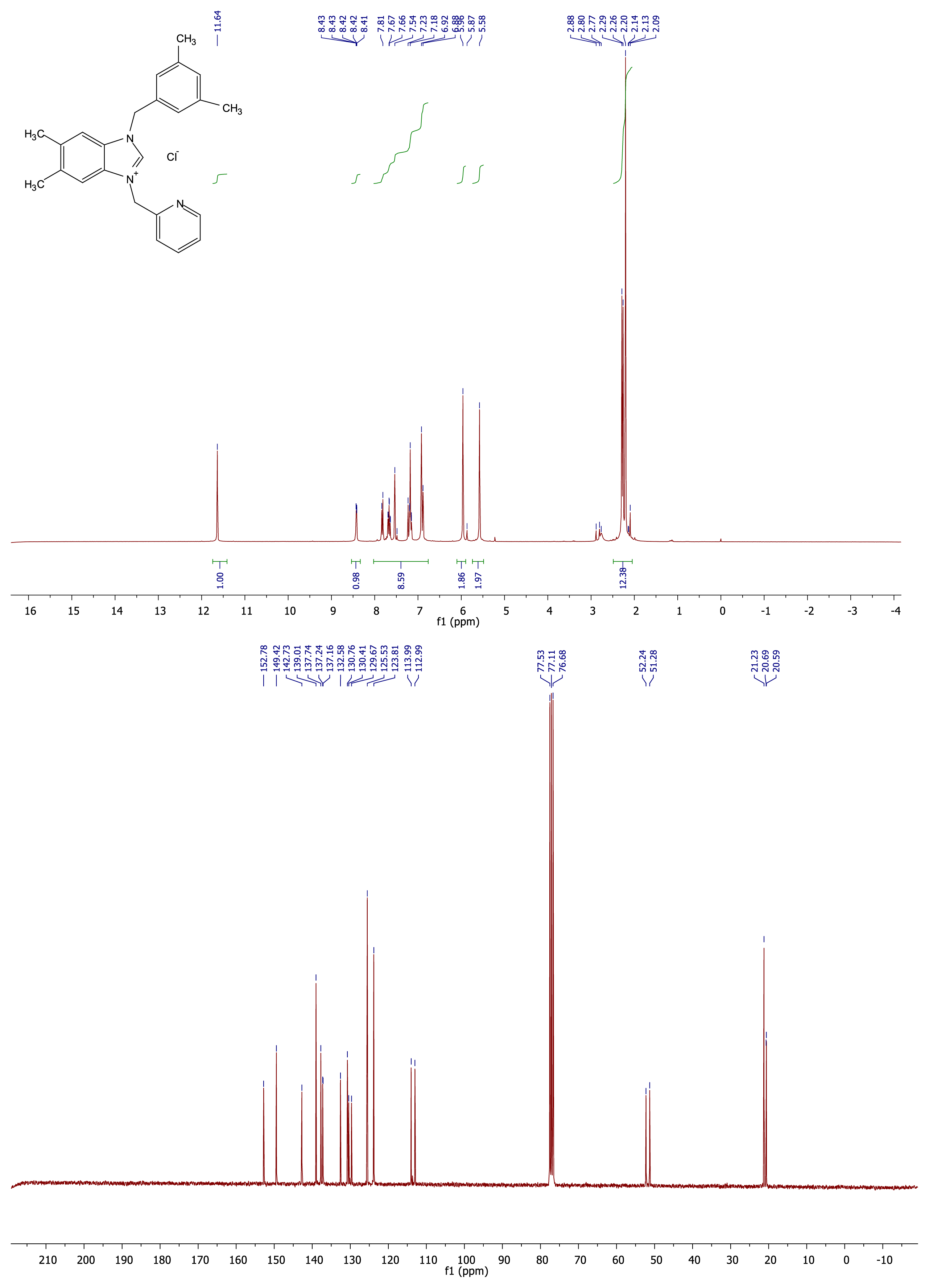

Supplement: Figure S3 — The 1H NMR and 13 NMR spectra of 2b. [file turkjchem-46-4-1097s3.tif]

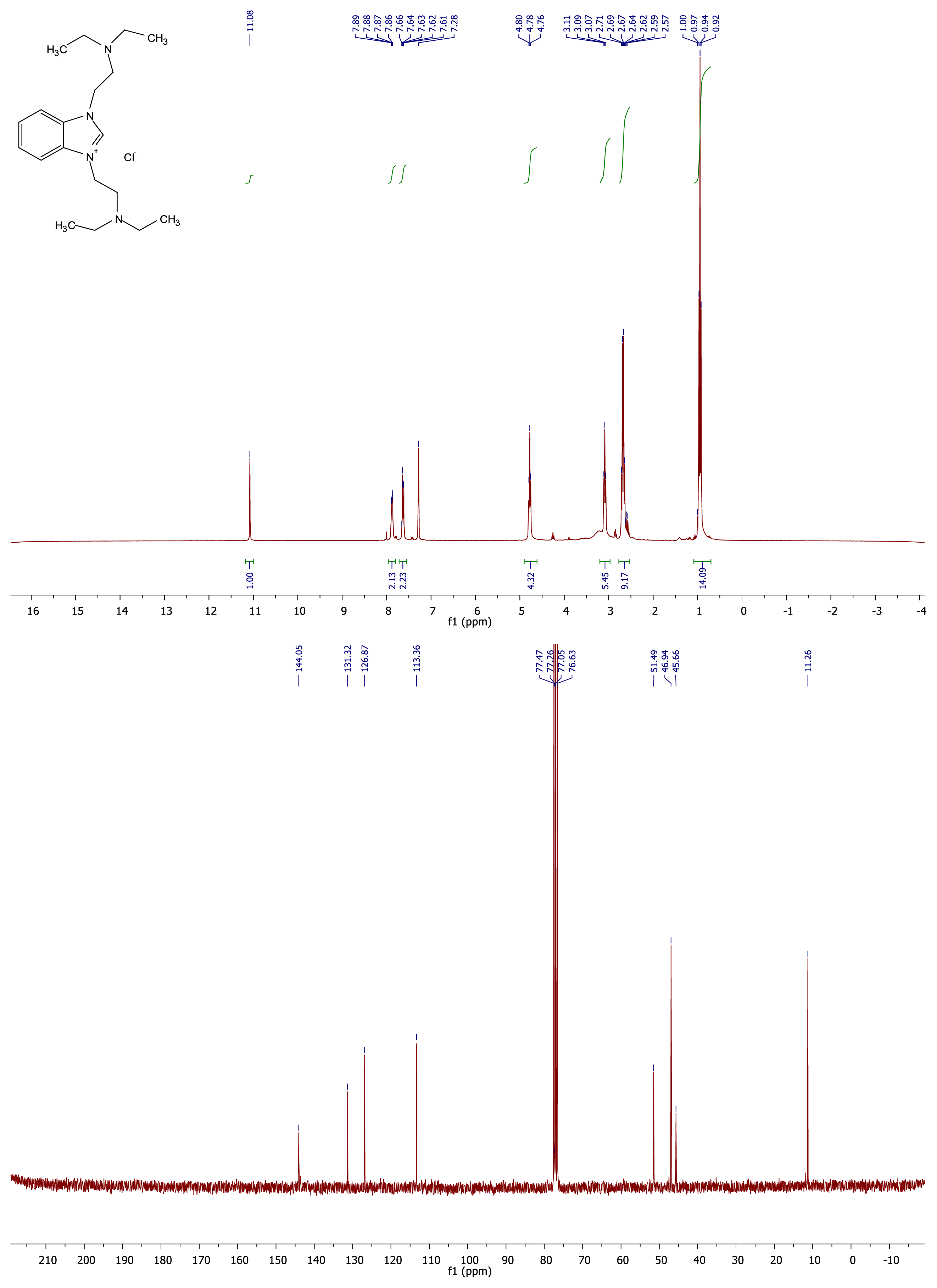

Supplement: Figure S4 — The 1H NMR and 13 NMR spectra of 2d. [file turkjchem-46-4-1097s4.tif]

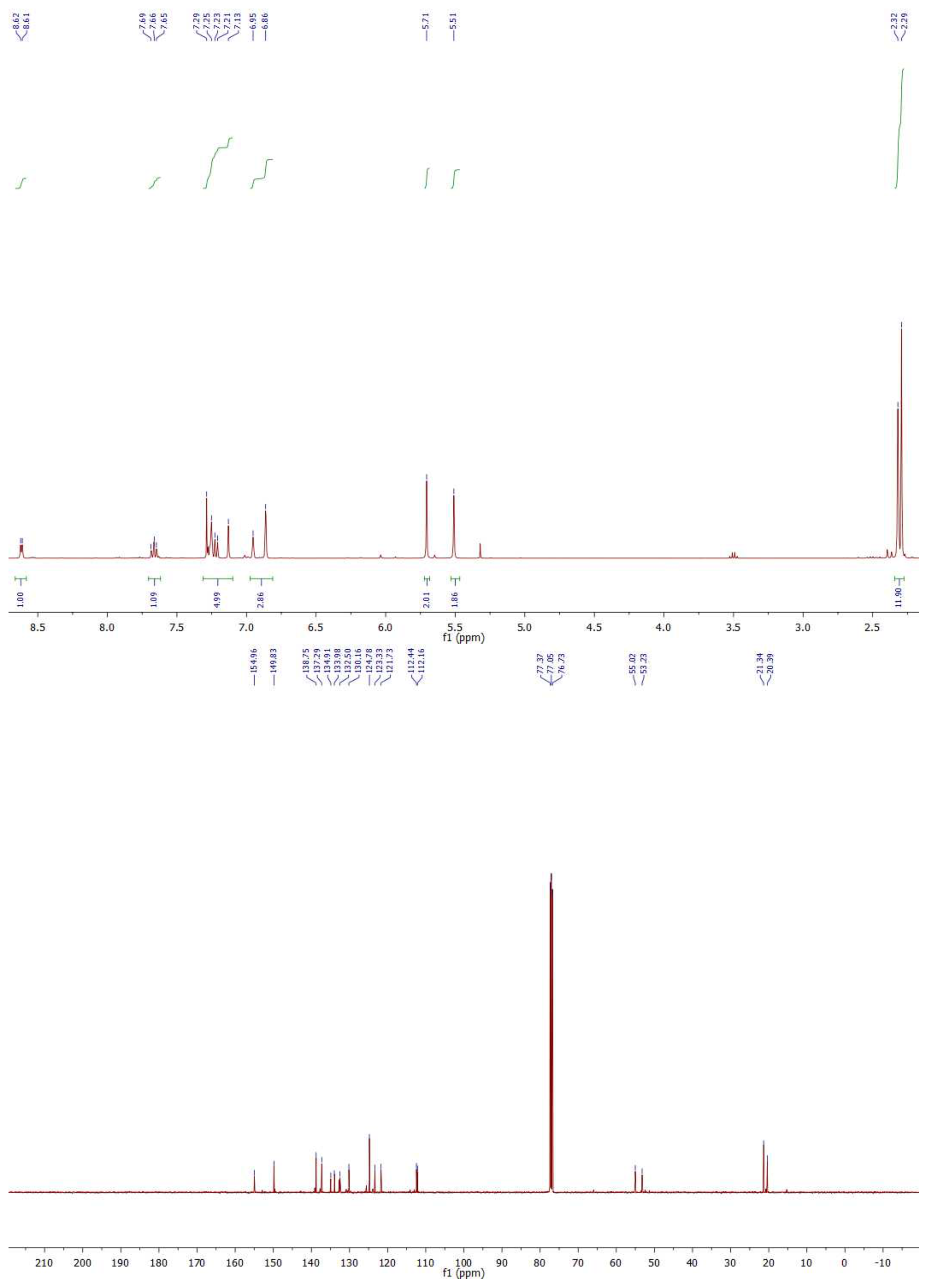

Supplement: Figure S5 — The 1H NMR and 13 NMR spectra of 3b. [file turkjchem-46-4-1097s5.tif]

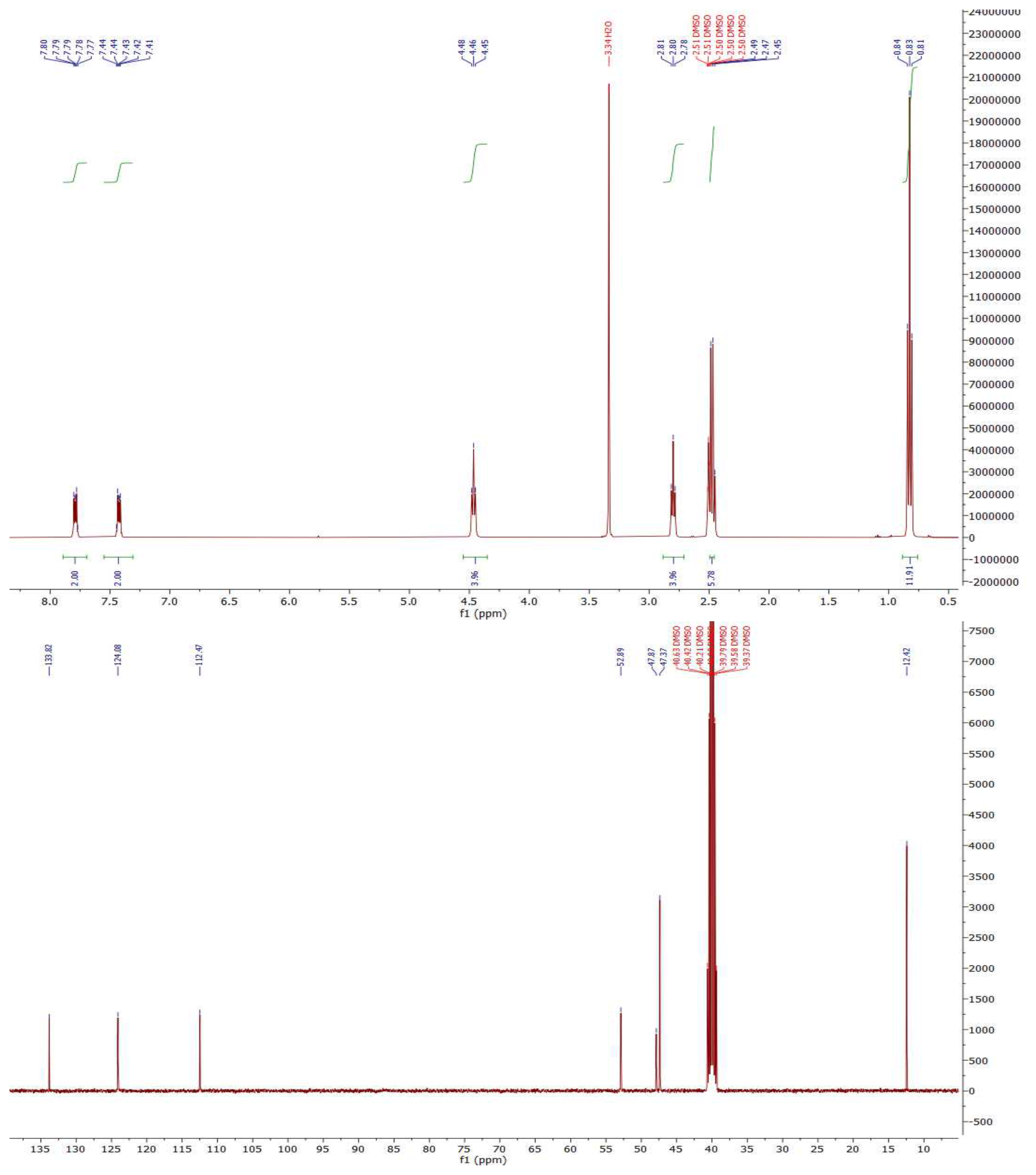

Supplement: Figure S6 — The 1H NMR and 13 NMR spectra of 3d. [file turkjchem-46-4-1097s6.tif]

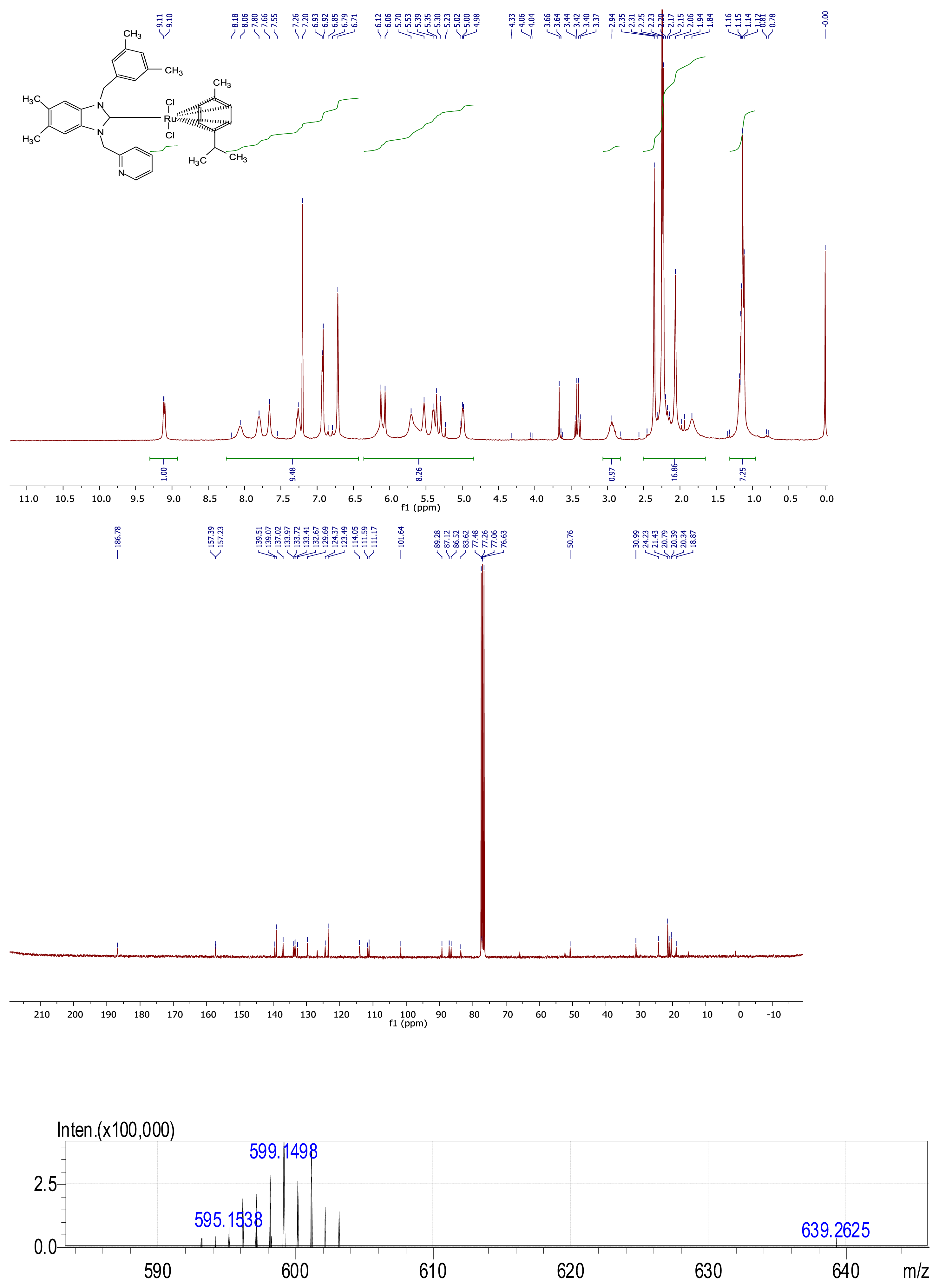

Supplement: Figure S7 — The 1H NMR, 13 NMR and HRMS spectra of 5b. [file turkjchem-46-4-1097s7a.tif]

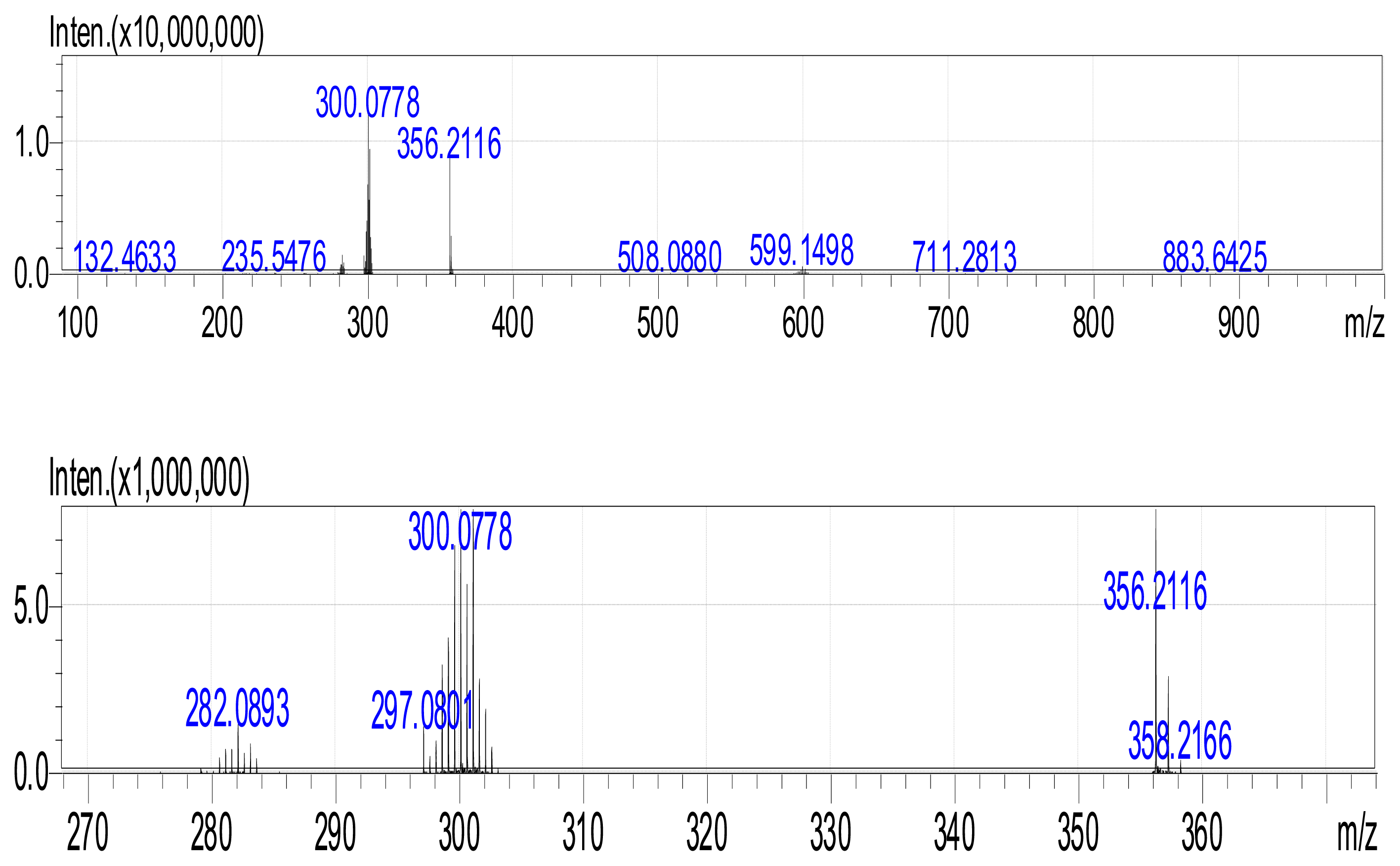

Supplement: Figure S7 — The 1H NMR, 13 NMR and HRMS spectra of 5b. [file turkjchem-46-4-1097s7b.tif]

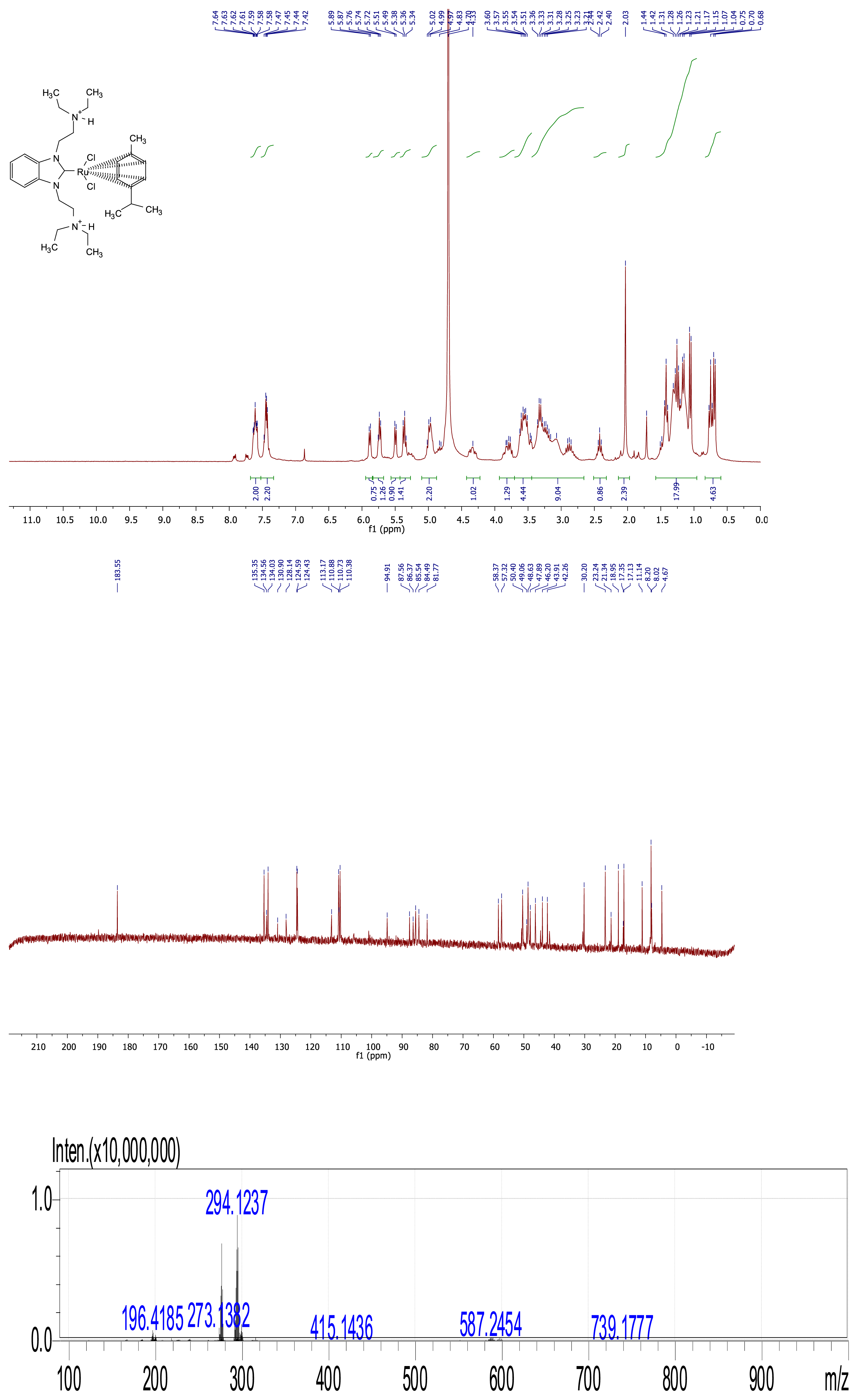

Supplement: Figure S8 — The 1H NMR, 13 NMR and HRMS spectra of 5d. [file turkjchem-46-4-1097s8a.tif]

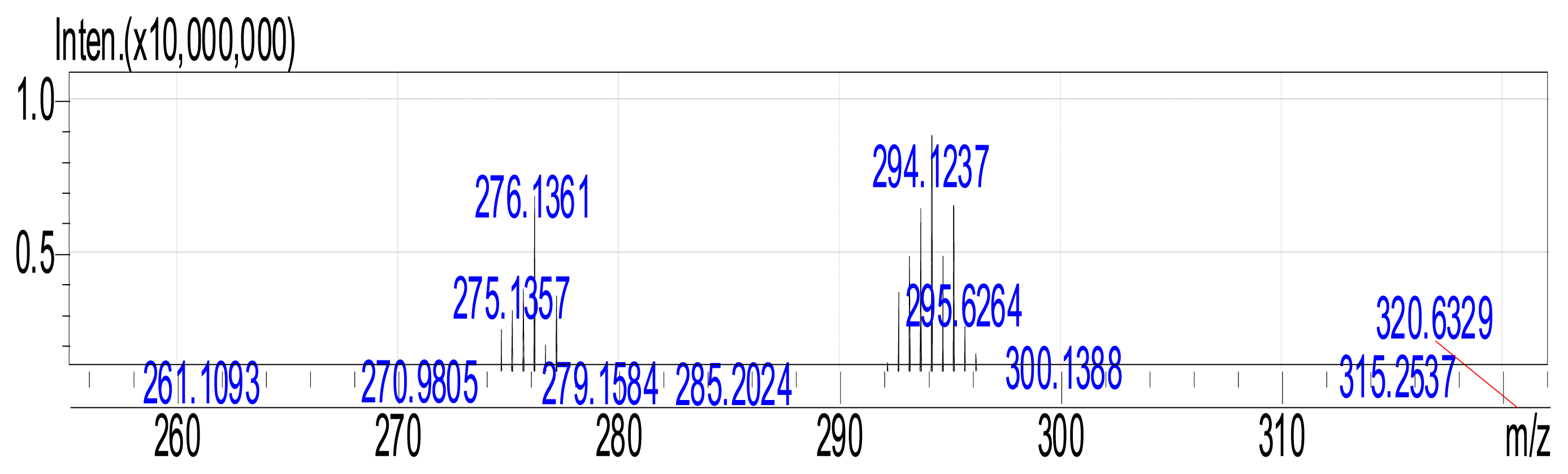

Supplement: Figure S8 — The 1H NMR, 13 NMR and HRMS spectra of 5d. [file turkjchem-46-4-1097s8b.tif]

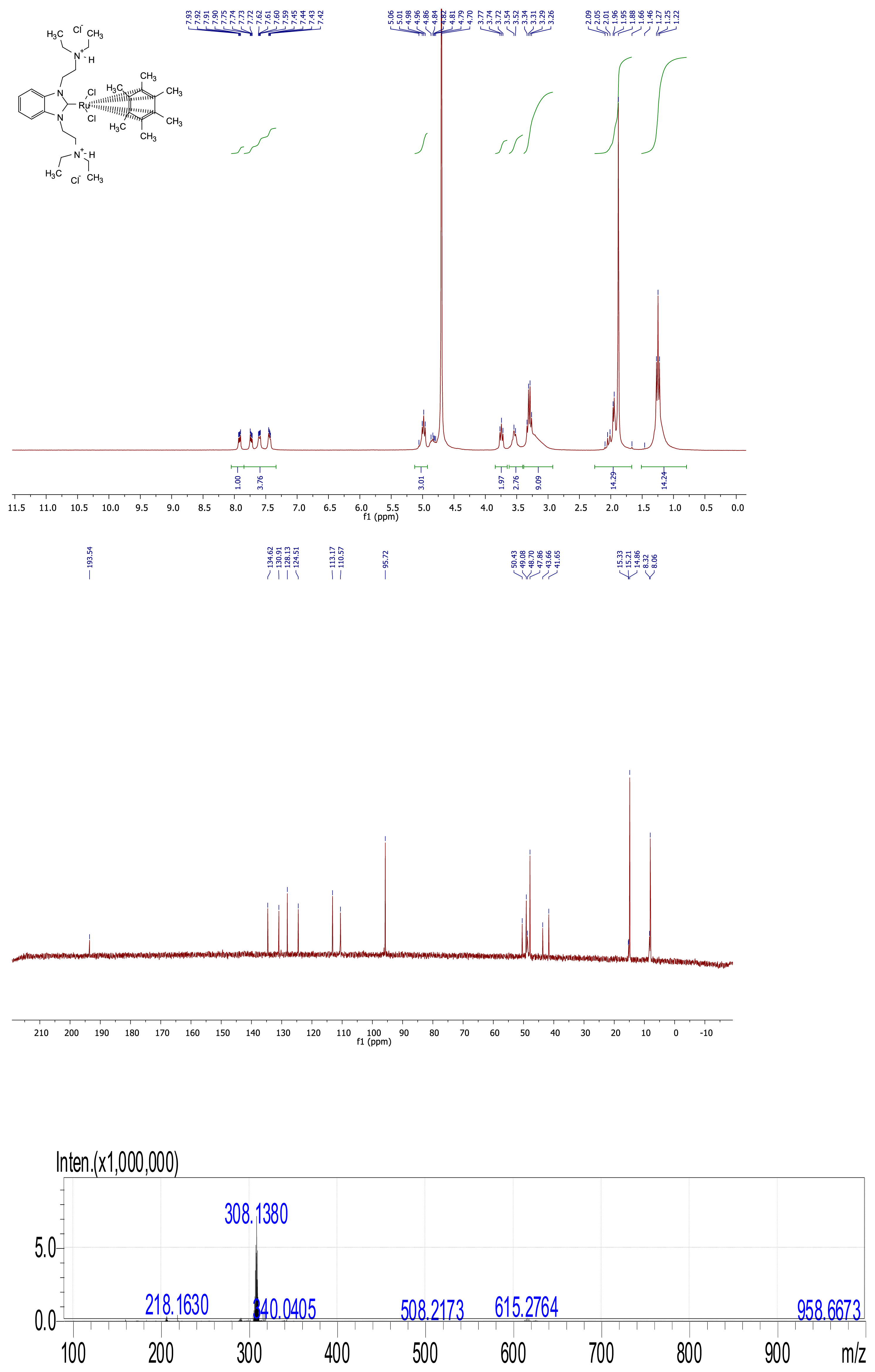

Supplement: Figure S9 — The 1H NMR, 13 NMR and HRMS spectra of 5f. [file turkjchem-46-4-1097s9a.tif]

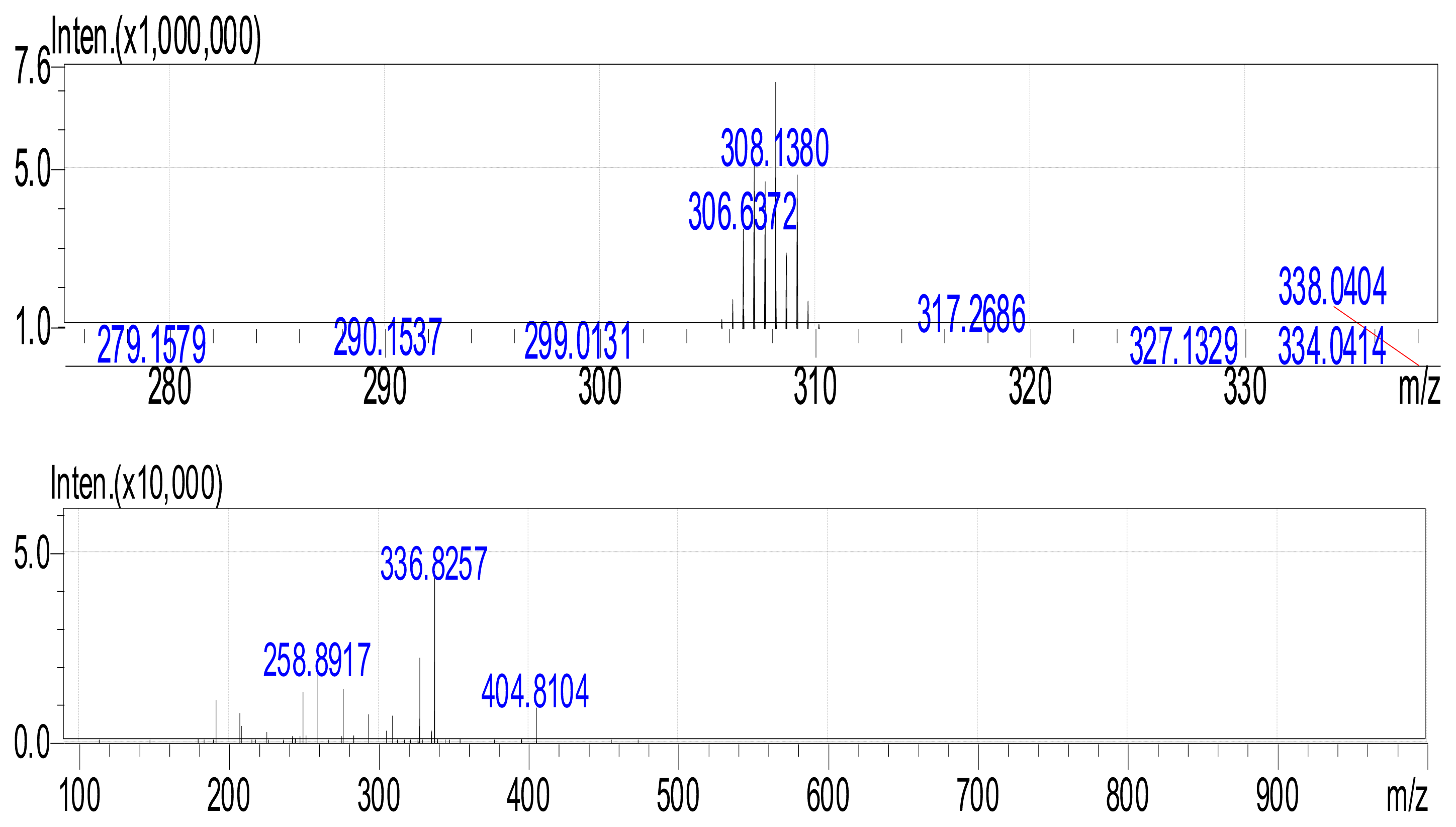

Supplement: Figure S9 — The 1H NMR, 13 NMR and HRMS spectra of 5f. [file turkjchem-46-4-1097s9b.tif]

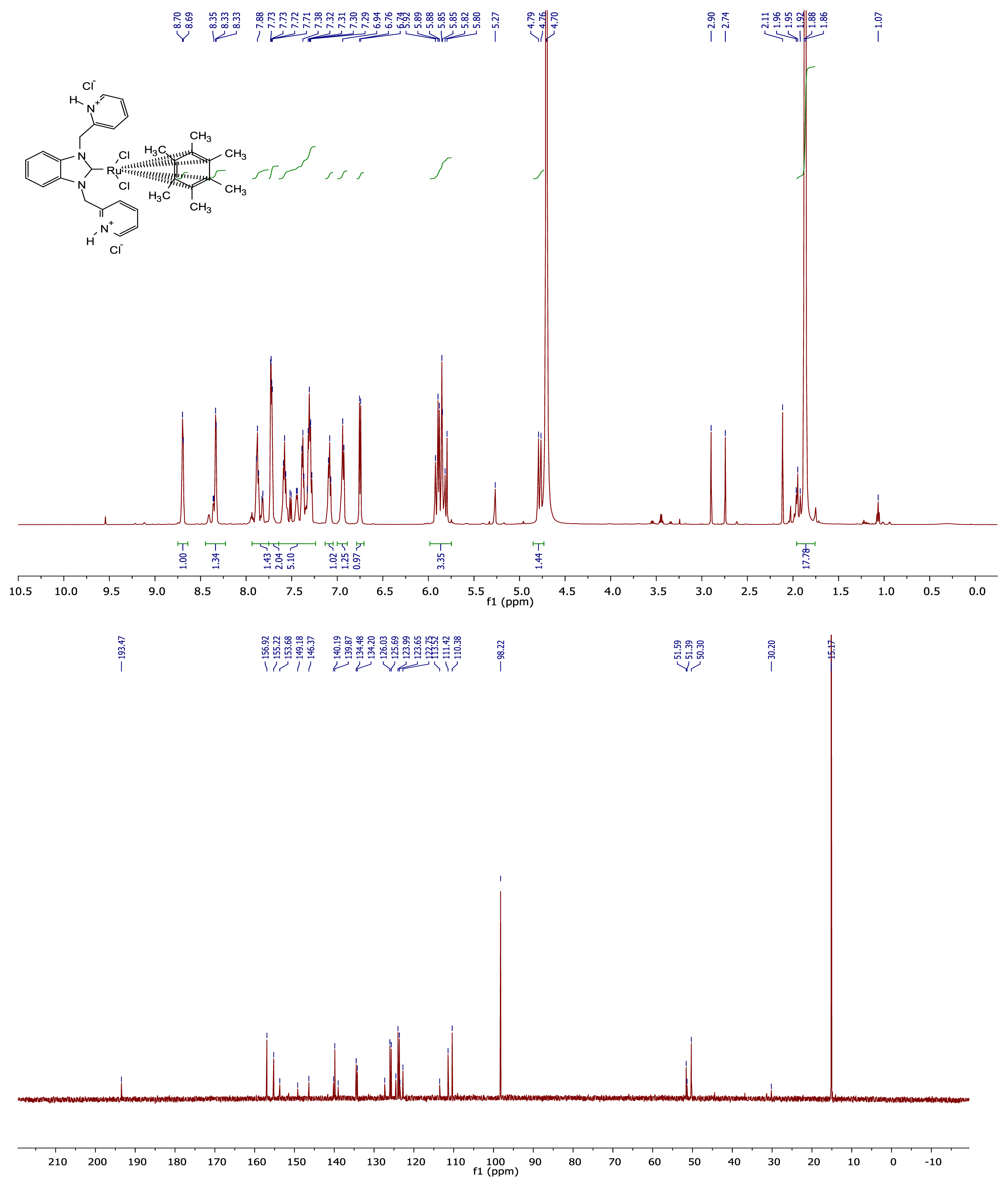

Supplement: Figure S10 — The 1H NMR and 13 NMR spectra of 5h. [file turkjchem-46-4-1097s10.tif]
